# Rewriting the script: gene therapy and genome editing for von Willebrand Disease

**DOI:** 10.3389/fgeed.2025.1620438

**Published:** 2025-09-22

**Authors:** Alastair Barraclough, Isabel Bär, Tirsa van Duijl, Karin Fijnvandraat, Jeroen C. J. Eikenboom, Frank W. G. Leebeek, Ruben Bierings, Jan Voorberg, Despoina Trasanidou

**Affiliations:** 1 Department of Pediatric Hematology, Emma Children’s Hospital, Amsterdam UMC, University of Amsterdam, Amsterdam, Netherlands; 2 Department of Hematology, Erasmus University Medical Centre, Rotterdam, Netherlands; 3 Molecular Hematology, Sanquin Research and Landsteiner Laboratory, Amsterdam University Medical Centre, Amsterdam, Netherlands; 4 Division of Thrombosis and Hemostasis, Department of Internal Medicine, Leiden University Medical Centre, Leiden, Netherlands

**Keywords:** gene therapy, VWD, endothelial cells, delivery, CRISPR-Cas, *in vivo* delivery

## Abstract

In recent years gene therapy has emerged as a powerful technology for treatment of a large variety of inherited disorders. With the FDA approval of *in vivo* gene therapy of hemophilia A and B using AAV-mediated transgene delivery to hepatocytes, the path towards a new treatment era seemed paved. Also, CRISPR-Cas based approaches have reached the clinic, as in the *ex vivo* treatment of hematopoietic stem cells for sickle cell disease and thalassemia patients. The question arises whether these innovative strategies will also be suitable for patients with von Willebrand Disease (VWD). Whilst *in* and *ex vivo* delivery to endothelial cells (ECs) has been demonstrated, and CRISPR-Cas9 gene editing has been successful in ECs, there are currently no gene therapy options available for VWD. The wide variety of pathogenic VWF mutations makes development of broadly applicable, cost-effective gene therapies challenging. While delivery of von Willebrand factor (VWF) as a therapeutic transgene would be optimal, the size of VWF challenges efficient delivery. Therefore, treatment of VWD requires targeted, personalized gene therapy; for instance by using the newest CRISPR-Cas technologies which can be tailored to facilitate alteration and restoration of various pathogenic VWD variants. This review describes the inherited bleeding disorder VWD and potential gene therapy approaches for management of the disease. Thereby we are exploring different CRISPR-Cas technologies and recent developments in the field. Moreover, we will discuss the ongoing advances of *in vivo* delivery systems, all with the scope on ECs.

## 1 Introduction on von Willebrand Disease (VWD)

### 1.1 The heterogeneous landscape of VWD

Von Willebrand Disease (VWD) is the most commonly inherited bleeding disorder, estimated to have a worldwide prevalence of 25.6 per million people in the general population (Leebeek and Eikenboom, 2016; Stonebraker et al., 2023). Patients with VWD have qualitative and/or quantitative abnormalities with von Willebrand Factor (VWF), a large multimeric protein that is pivotal during primary hemostasis. There are different types of VWD, with ranging severities. Type 1 VWD is the most common, where patients have reduced VWF in circulation and a mild bleeding phenotype (Atiq et al., 2023). If the reduced amount of VWF is due to enhanced clearance, it is described as type 1C VWD. Type 2 VWD is characterized by a qualitative deficiency of VWF and can be further classified into various subtypes: A, B, M, and N (Table 1). The qualitative impairment spans from insufficient multimerization of VWF dimers into its hemostatically active mature VWF multimer, to impaired binding and susceptibility to other proteins involved in maintaining hemostasis (Daniel et al., 2024; Jacobi et al., 2012; Maas et al., 2021; Othman and Favaloro, 2021). Type 3 is the rarest but most severe form, where patients have close to undetectable VWF levels in circulation and experience a severe bleeding phenotype (Eikenboom, 2001; van Kwawegen and Leebeek, 2024).

**TABLE 1:** Summary of von Willebrand disease sub-types, disease mechanism, and treatment.

VWD type	Autosomal inheritance	Common types of mutation	Common localization of variants	Mechanism of VWF abnormality	VWD phenotype	Common treatment
1	Dominant	Missense, Null allele	Across VWF gene	Reduced VWF synthesis/ secretion, no protein synthesis from one allele	Reduced VWF levels in plasma	DDAVP
1c	Dominant	Missense	Across VWF gene	Increased clearance of VWF	Reduced VWF levels in plasma	DDAVP
2A	Dominant	Missense	D1 D2 (VWFpp), D3, A2, CK domains	Decreased formation of HMW VWF due to defective dimerization/ multimerization, increased cleavage by ADAMTS13	Reduced platelet interaction due to reduced HMW VWF	DDAVP, VWF/ factor concentrates
2B	Dominant	Missense	A1 domain	Increased and spontaneous binding to platelet GPIb	Decreased VWF in circulation due to clearance of VWF-platelet aggregates	VWF/ factor concentrates
2N	Recessive	Missense, Null allele + missense	D’ D3 domains	Decreased FVIII binding	Increased bleeding due to limited VWF chaperoning of FVIII (reduced FVIII half life)	DDAVP
2M	Dominant	Missense	A1 and A3 domains	Reduced affinity of VWF for platelet GPIb or collagen	Increased bleeding due to reduced platelet adhesion	DDAVP
3	Recessive	Null allele	Across VWF gene	Nonsense mediated decay	No VWF detectable in circulation	VWF/ factor concentrates

**FIGURE 1:**
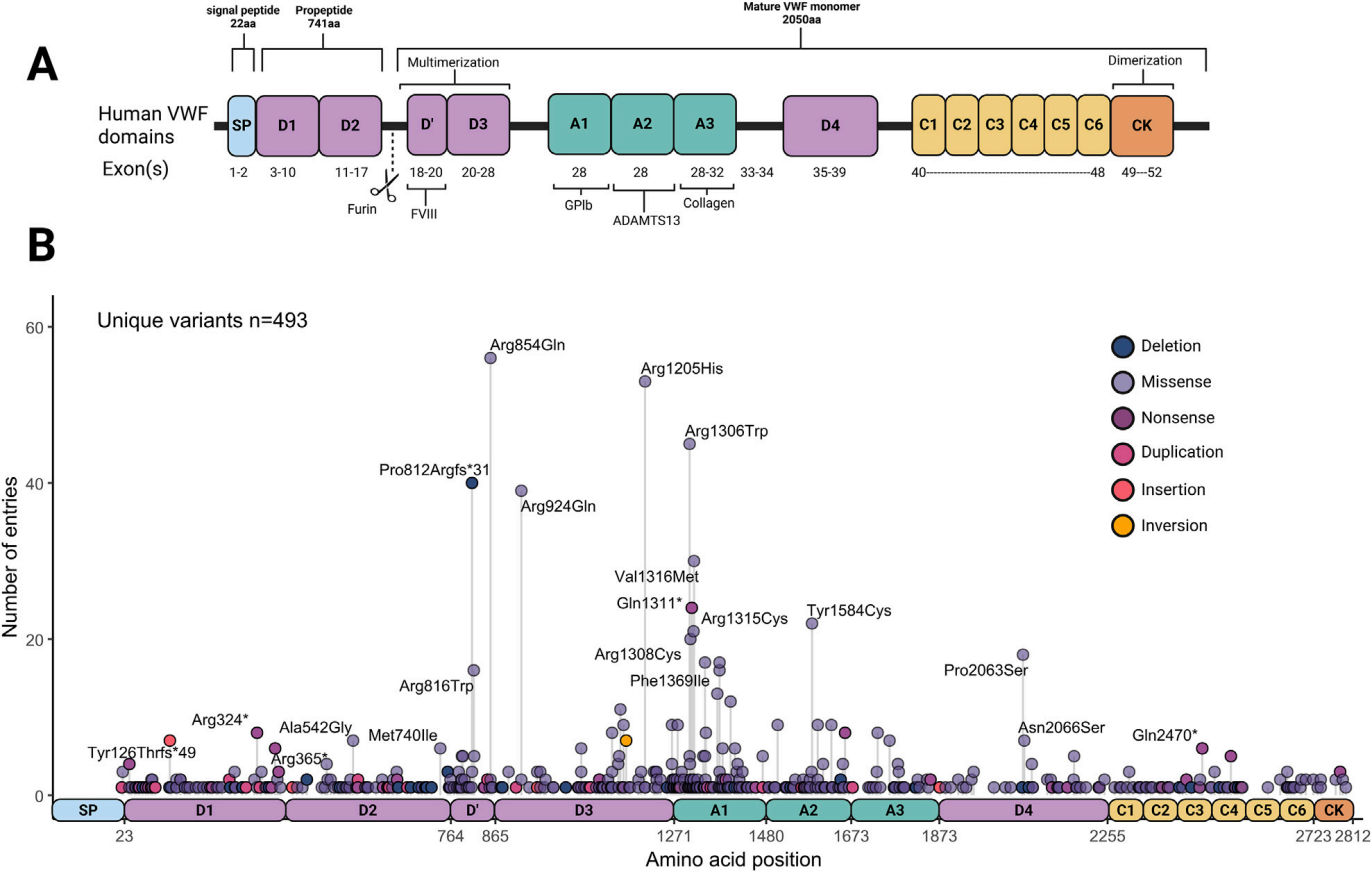
Overview of von Willbrand Factor (VWF) domains, and variant distributions. **(A)** Von Willebrand Factor domains and binding sites. **(B)** Distribution and frequency of von Willebrand Factor variants curated in the Leiden Open Variation Database (LOVD) (Accessed and data retrieved on 8-1-2025). Created in BioRender.

The different types of VWD often arise from mutations impacting different domains of VWF (Figure 1A). Currently, more than 750 unique pathogenic VWF mutations are known, leading to a range of bleeding complications including mucocutaneous bleeding, heavy menstrual bleeding, joint bleeds, gastrointestinal bleeding and bleeding during surgery (de Jong and Eikenboom, 2017; Weyand and Veronica, 2021). The Leiden Open Variation Database (LOVD) alone reported a list of 505 *VWF* variants that were found in VWD patients in the clinic, of which ∼450 were missense and nonsense variants (Seidizadeh et al., 2023). Utilizing those variants from the LOVD database as an example, Figure 1B illustrates the distribution of VWF variants. Variants resulting in type 1 and 3 VWD can be found throughout *VWF*. In contrast, type 2 VWD variants are often located around the A1 domain, affecting the binding to platelets and/or collagen, or around the D’D3 or CK domains, impacting multimerization. More *VWF* variants have been reported by various studies and can be found in additional databases such as ClinVar, GnomAD, genome browser, human gene mutation database (HGMD) and more (UCSC, 2025; gnomAD, 2025; NCBI, 2025; HGMD, 2025). Although the prevalence of VWD subtypes varies per database and cohort, multiple studies have shown that type 1 VWD is by far the most common form, accounting for around 60%–85% of VWD cases. Type 2 VWD follows as the second most common with a prevalence of 15%–45% (Soucie et al., 2021; Seidizadeh et al., 2023). Orphanet estimates the prevalence of type 1 VWD worldwide to be 1–5/ 10,000, and the prevalence of type 2 VWD worldwide to be 1–9/1,000,000 (Orphanet, 2025a; Orphanet, 2025b). Type 3 VWD remains the rarest form of the disease.

Even though in around 30% of patients no pathogenic mutation in *VWF* could be identified, VWD is generally considered a monogenic disease. Detecting the location of a disease-causing variant within *VWF* may indicate the impaired domain and binding site of the protein, thus implying the underlying molecular mechanism impairing VWF. However, the majority of variants that are found within *VWF* are not sufficiently characterized and are thus classified as variants of uncertain significance (VUS). To shed light on this, studies have sequenced large patient populations and performed *ex vivo* characterization experiments to explore which VWD variants are truly pathogenic (Atiq et al., 2022; Bär et al., 2025; Christopherson et al., 2022; 2024; James et al., 2006; Laan et al., 2024; Tosetto et al., 2020; Vangenechten et al., 2022).

### 1.2 Inheritance of VWD

VWD is predominantly inherited as an autosomal dominant disease, with the majority of VWF variants being heterozygous missense mutations. This results mostly in VWD type 1 and 2 subtypes, while type 3 and type 2N often derive out of homozygous null mutations or compound heterozygous and are therefore rarer forms of VWD with a typically recessive inheritance pattern. The prevalence for type 3 lays around 1:250,000 to 1:1,000,000 but is challenging to determine because of the increased prevalence in regions where consanguinity is more common (Nichols et al., 2008).

### 1.3 The star of primary hemostasis: von Willebrand Factor (VWF)

**FIGURE 2:**
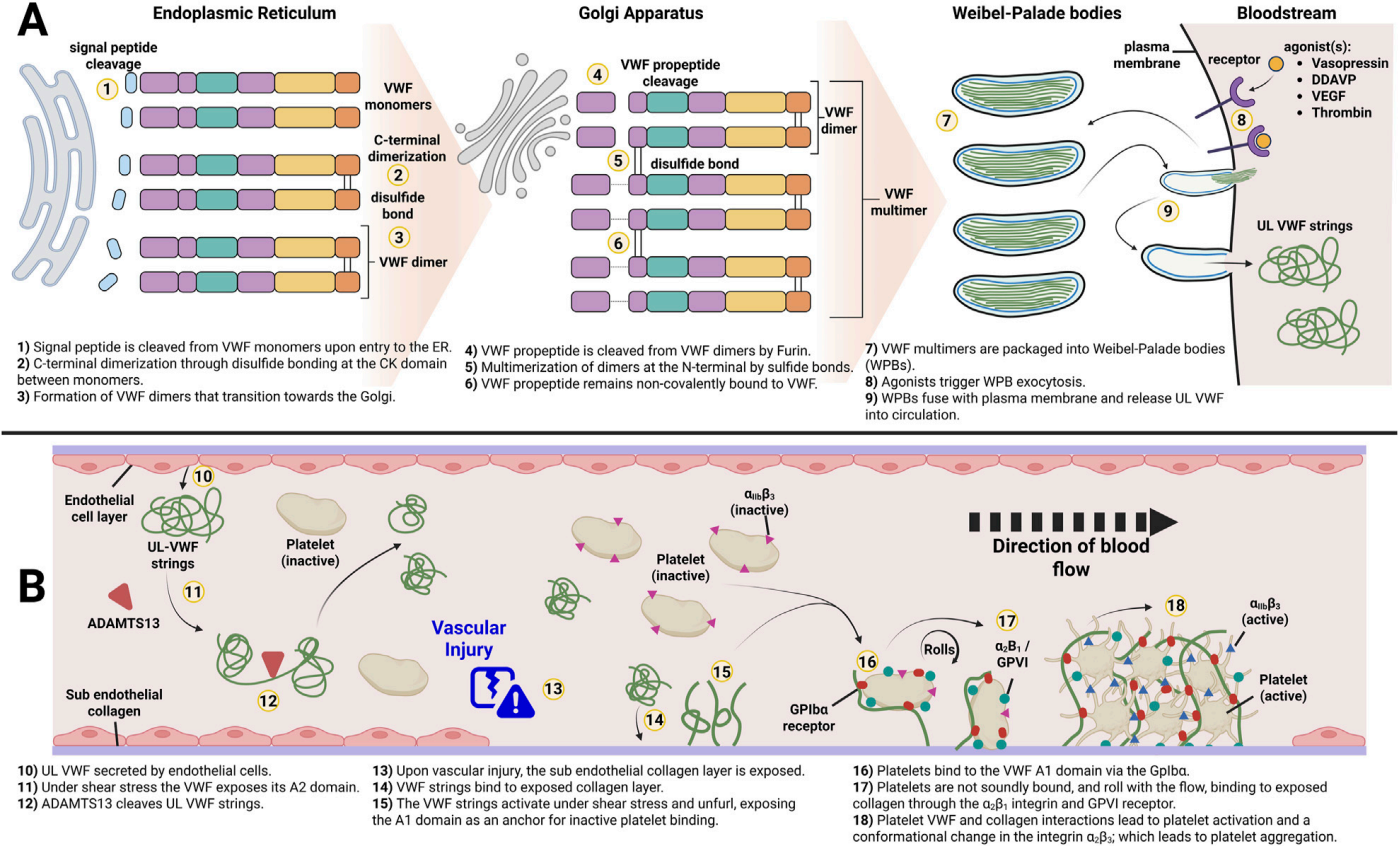
Overview of von Willebrand Factor biosynthesis and its role in primary hemostasis. **(A)** Key processing steps by endothelial cells of von Willebrand Factor to form Weibel-Palade Bodies. **(B)** Function of HMW secreted from WPBs. Adapted from Leebeek and Eikenboom, 2016. Created in BioRender.

In VWD there can be quantitative or functional deficiency of VWF: a large multimeric protein that is produced by endothelial cells (EC) and megakaryocytes. Long VWF strings are primarily stored in Weibel-Palade bodies (WPB) of ECs and α-granules of platelets, respectively (Lenting et al., 2015). In ECs after synthesis (Figure 2A), VWF is translocated to the endoplasmic reticulum (ER) where the VWF signal peptide is removed, and C-terminal dimerization occurs between VWF monomers via the CK domains (Hordijk et al., 2024). The dimers are subsequently shuttled to the Golgi, where Furin cleaves the VWF propeptide domain (VWFpp) off the dimers, allowing for their multimerization via the N-terminal D′ D3 domains. Importantly, the VWFpp remains non-covalently bound to the VWF multimers which is essential for trafficking and packaging of VWF multimers into WPBs (Haberichter, 2015). These organelles store ultra large (UL) and high molecular weight (HMW) VWF multimers which are secreted into the bloodstream upon endothelial activation. At the same time, low molecular weight (LMW) VWF is constantly secreted by the ECs in a process called constitutive secretion. The third secretion pathway of VWF is the unstimulated fusion of WPBs and the release of its content during basal secretion (Silva et al., 2016). The basally secreted VWF is considered the main contributor to stable VWF levels in plasma. When secreted, the UL and HMW VWF strings undergo cleavage by ADAMTS13, resulting in a distinct multimeric pattern of different sizes (Giblin et al., 2008; Zhang et al., 2009). Biosynthesis of VWF in megakaryocytes also results in the formation of HMW VWF multimers that are eventually stored in tubular sub-compartments of platelet α-granules (Karampini et al., 2020).

In plasma, VWF acts as a chaperone for FVIII, protecting it from clearance and proteolytic cleavage (Pipe et al., 2016). At sites of vascular injury, VWF binds to the exposed sub-endothelial collagen layer through its A3 domain, anchoring itself and unfolding to expose its platelet binding sites (Romijn et al., 2001). The unfolded VWF strings provide a platform for platelets to bind to the A1 domain through their glycoprotein Ibα (GPIbα) receptor, initiating the formation of a platelet plug (Lenting et al., 2024; Figure 2B). This completes the process called primary hemostasis (Karampini et al., 2020). Due to the versatile role of VWF and its complex structure including several binding sites, the variety in VWD phenotypes is not surprising. Deficiency of VWF impacts other proteins involved in coagulation and the treatment and prevention of bleeds caused by faulty or missing VWF is challenging.

### 1.4 Current treatment of VWD

The plethora of pathogenic variants and disease mechanisms complicates treatment of VWD tremendously. In addition, the lack of knowledge about the disease-underlying causes currently prevents personalized treatment in the clinic. Current treatment aims to increase VWF and FVIII plasma concentrations on demand in case of a bleeding event or before and after interventions. A common treatment approach for VWD patients, especially type 1, is to increase circulating VWF and FVIII levels by stimulating release of stored VWF and FVIII from ECs. This is achieved via the administration of desmopressin (DDAVP), a synthetic version of the hormone vasopressin, which interacts with the V2R receptor of ECs to stimulate VWF release (Phua and Erik, 2019). However, not all VWD patients respond to DDAVP and DDAVP administration is contraindicated in patients with type 2B VWD, due to increased risk of thrombocytopenia as their VWF has an increased affinity for platelets (Laan et al., 2025). Also, patients with type 3 VWD do not benefit from DDAVP, since there is no stored VWF that can be released by ECs (Connell et al., 2021). According to the ASH ISTH NHF WFH 2021 VWD guidelines, DDAVP has to be periodically tested in patients for which this could be a viable option, and treatment should be adjusted based on the results of these trials (James et al., 2021).

An alternative for DDAVP for unresponsive patients or in major surgery where DDAVP is not sufficient, is replacement therapy with VWF (FVIII) factor concentrates. Replacement therapy is used in the management of acute bleeds and in severely affected VWD patients to prevent bleeding in the long-term. The latter patients receive factor concentrate 2–3 times per week intravenously (so-called prophylaxis). Plasma-derived VWF concentrates and recombinant VWF (rVWF) are both effective to stop and prevent bleeding (Gill et al., 2015; Leebeek et al., 2022). The antifibrinolytic agent tranexamic acid is mostly used as an adjunctive therapy to DDAVP or VWF concentrates (Laffan et al., 2014).

Taken together, there are effective therapies in place for patients with VWD. However, VWF concentrates need to be administered intravenously by a healthcare professional and therefore require a hospital visit. Especially in severely affected patients, like VWD type 3 patients on long term prophylaxis, regular hospital visits decrease their quality of life. In addition, concentrates are costly and represent a monetary burden for society due to the life-long treatment need. The current treatment is therefore sub-optimal and a durable treatment approach would be ideal to reduce the burden of disease in patients with VWD; allowing them to engage more in their daily activities with reduced concern. Therefore, alternative and innovative therapies are needed to optimize treatment of VWD. In the following paragraphs we will discuss the suitability of different gene therapy approaches for personalized treatment of VWD.

## 2 Silencing of mutant VWF in heterozygous VWD patients using small interfering RNA (siRNA)

**FIGURE 3:**
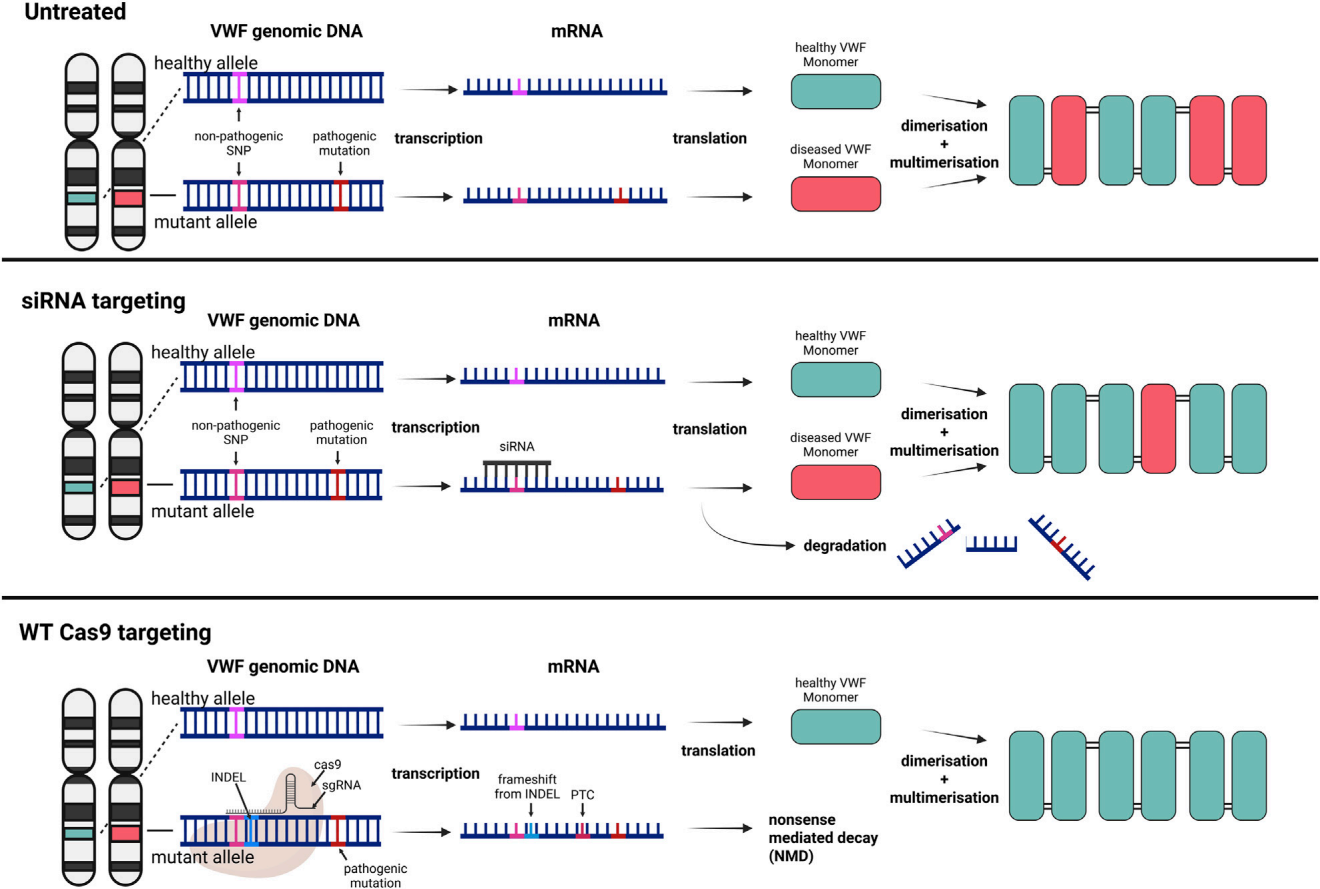
Broad targeting gene therapy strategies for dominant negative VWD variants to increase the ratio of healthy to diseased VWF subunits. Top) Untreated VWD results in VWF multimers consisting of healthy and diseased subunits. Middle) Selective siRNA targeting of a benign SNP on the mRNA originating from the mutant allele results in degradation of the transcript and a higher ratio of healthy to mutant VWF subunits. Bottom) The same targeting approach of a benign SNP, but the Cas9 directly targets the genomic DNA and induces a frameshift and premature termination codon (PTC). The knockout of the mutant allele results in sole expression of healthy VWF monomers. Created in BioRender.

RNA interference (RNAi) is a mechanism whereby mRNA is degraded through targeting by complementary short interfering RNA (siRNA) molecules. In hemophilia patients, siRNA targeting antithrombin has been studied extensively with the aim to restore the hemostatic balance. Patients treated with this siRNA showed a normalization of thrombin generation and reduction of the number of bleedings (Srivastava et al., 2021). A similar approach can be used in heterozygous VWD patients whereby siRNA is targeting the mutated, disease-causing allele. As mentioned above, around 33% of VWF mutations lead to a qualitative impairment, resulting in type 2 VWD (Seidizadeh et al., 2023). In human umbilical vein endothelial cells (HUVECs), siRNA-mediated knock-down of VWF can achieve 90% reduction at the transcript level (Furini et al., 2023; Seidizadeh et al., 2023). De Jong et al. enhanced this approach by encapsulating siRNA targeting mutant VWF mRNA in lipid nanoparticles (LNPs), resulting in allele-selective VWF degradation in a dominant negative type 2A VWD model (Figure 3). Through this approach, they demonstrated *ex vivo* resolution of VWF ER retention in a VWD type 2A phenotype using patient-derived venous endothelial colony-forming cells (ECFCs) (De Jong et al., 2020). The siRNA was designed to target a non-pathogenic, commonly occurring heterozygous single-nucleotide polymorphism (SNP) that co-segregated with the pathogenic mutation encoding for the dominant negative VWD variant. This concept was then further explored in mice, by crossing two different mouse strains, B6 and 129S, to create a heterozygous B6.129S model. This model mimicked a heterozygous scenario where the siRNA could discriminate between a single benign SNP present in the two VWF alleles (Jongejan et al., 2023; 2024). Following this proof of concept, Linthorst et al. investigated whether this approach could be extended to a heterozygous VWD type 2B mouse model (Linthorst et al., 2024). The resulting selective reduction of mutant VWF protein, the improved multimeric VWF pattern, and the normalized bleeding times in two-thirds of the heterozygous VWD 2B mice highlight the potential of this personalized therapeutic treatment strategy. The targeting of a heterozygous SNP instead of the disease-causing variant also allows for a broader application, independent of the pathogenic VWD mutation. De Jong et al. identified 4 common SNPs with a minor allele frequency of approximately 0.3 in the general Caucasian population. Based on these frequencies, they calculated that a person has a 74% probability of being heterozygous for at least one of these SNPs (De Jong et al., 2020). In another study, WT and a dominant negative VWF variant plasmid that impacts multimerization were co-injected into VWF^−/−^ mice. The VWF variant was then counteracted by a follow-up siRNA injection; demonstrating an improvement in multimerization on the following day (Campioni et al., 2021). This elegant approach may potentially be used for successful treatment of a subset of patients with dominant negative type 2 VWD. However, siRNAs do not provide a permanent solution for patients with VWD but require regular re-administration (Linthorst et al., 2025). How long the siRNAs would be functional *in vivo* and alleviate the disease phenotype still needs to be evaluated in large animal models and humans.

## 3 CRISPR-cas technologies

**FIGURE 4:**
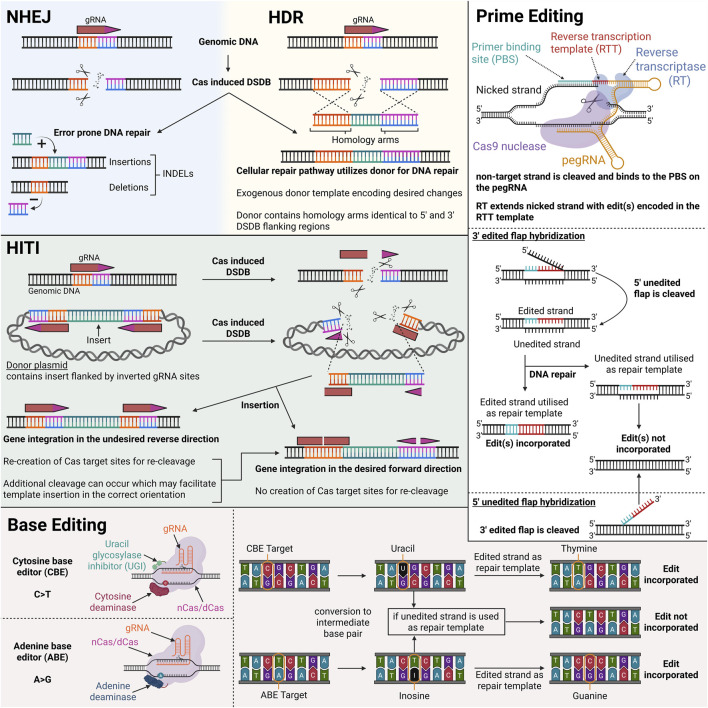
Illustration of different CRISPR-Cas modalities and their mechanisms of genomic DNA modification. Created in BioRender.

Clustered regularly interspaced short palindromic repeats (CRISPR) and CRISPR-associated (Cas) proteins are adaptive immune systems protecting their prokaryotic host against invading genetic elements. Since 2012, various Cas nucleases have been repurposed as next-generation genetic engineering tools, replacing previous time-consuming and costly technologies such as zinc-finger nucleases (ZFNs) and transcription activator-like effector nucleases (TALENs). CRISPR-Cas systems are categorized into two classes, seven types and several subtypes. Class 2 systems have taken current genome editing applications by storm, due to their simple architecture of a single effector protein (Cas9, Cas12, and Cas13), in contrast to the multi-protein effector complex nature of Class 1 systems. Nowadays, the most widely used Class 2 nucleases are the type II-A Cas9 from *Streptococcus pyogenes* (SpCas9) and the type V-A Cas12a from *Lachnospiraceae* bacterium (LbCas12a) or *Alicyclobacillus acidoterrestris* (AsCas12a). Cas9 and Cas12a nucleases require a small guide RNA (gRNA) molecule to bind to a target DNA sequence (protospacer) flanked by a short, conserved motif (protospacer adjacent motif; PAM) and create a double-strand DNA break (DSDB) (Jinek et al., 2012; Zetsche et al., 2015) Several other naturally occurring Cas9 or Cas12 orthologues and engineered variants thereof have also been harnessed, enriching the current CRISPR-Cas toolbox (Acharya et al., 2019; Agudelo et al., 2020; Chatterjee et al., 2018; Fedorova et al., 2020; Harrington et al., 2017; Hu et al., 2020; Lee et al., 2016; Liu et al., 2019; Müller et al., 2016; Ran et al., 2015; Trasanidou et al., 2023; Wang et al., 2023; Wu T. et al., 2023; Wu et al., 2022). Below is an overview of how different CRISPR-Cas technologies can be employed for treatment and/or correction of VWD, with Figure 4 illustrating their mechanisms.

### 3.1 DSDB-based gene editing: non-homologous end joining (NHEJ) and homology directed repair (HDR)

There are two main pathways to repair a DSDB generated by class 2 Cas nucleases: non-homologous end joining (NHEJ) and homology-directed repair (HDR). The NHEJ approach exhibits high efficiency due to its independence from cell cycle and the continuous editing at the recognition site of the gRNA until random insertions and/or deletions (INDELs) are achieved, which usually prevent further Cas:gRNA binding. In contrast, HDR introduces precise genetic modifications and requires a DNA repair template and dividing cells. The presence of an exogenous DNA template tricks the cell to utilize the exogenous template for repair, rather than the sister chromatid during homologous recombination (HR) (Liao et al., 2024). Except from the aforementioned restrictions of HDR, a strong DSDB-induced NHEJ background is also usually observed (Chapman et al., 2012; Cox et al., 2015). Confirmation of CRISPR-induced modifications in the context of VWD can be achieved through both genotypic and phenotypic analyses. Next-generation sequencing enables precise detection of molecular alterations introduced by CRISPR, while phenotypic validation such as improved VWF synthesis observed via *ex vivo* confocal microscopy of patient-derived ECFCs or changes in VWF plasma levels *in vivo* could further substantiate successful gene editing.

#### 3.1.1 Non-homologous end joining (NHEJ)

In mammalian cells, the DSDB is mainly repaired via the error prone NHEJ repair pathway; resulting in frameshifts and premature termination codons (PTC) due to small INDELs. This approach has been utilized by Schillemans et al., employing SpCas9 in a lentiviral system in cord-blood (CB) ECFCs to generate clonal VWF knock-out (KO) lines (Schillemans et al., 2019). With this study the effectiveness of Cas9 in ECFCs has been proven and paved the way for further *ex vivo* patient-derived ECFC studies.

Following the same rationale as allele-selective siRNA targeting, the CRISPR-Cas technology may be exploited for the selective disruption of mutant VWF alleles harboring pathogenic variants (Figure 3). In an ongoing study we observed a phenotypic rescue in type 2A and type 2B VWD patient-derived venous ECFCs by targeting a common heterozygous SNP located on the same allele as the pathogenic variant (Bär et al., 2024). A key consideration of CRISPR-based approaches for selectively silencing pathogenic alleles of VWF lies in the potential for off-target activity. In particular, insufficient discrimination between the mutant and wild-type alleles by the gRNA can lead to unintended silencing of the functional allele. Such off-target effects could exacerbate the clinical phenotype of VWD, rather than ameliorate it, by further reducing the levels of functional VWF. Simultaneously, selective targeting of ECs needs to be ensured to prevent genome-wide off-targeting of the CRISPR construct. As an additional safety measure, an EC specific promoter could be leveraged to ensure limited expression in undesired tissue *in vivo*. Nevertheless, this targeting strategy is a promising first step towards a permanent rescue of heterozygous VWD types, providing a high allele-selectivity, and limiting disruption of healthy alleles. Whilst the approach of allele-selective targeting, whether with siRNAs or CRISPR-Cas technology, show promising results for alleviating the dominant-negative effect of mutant VWF, the risk of haploinsufficiency remains, which has previously been reported for VWD type 1 (Desch et al., 2013; Sadler et al., 2022). Theoretically, severely affected heterozygous VWD type 2 patients could transition to a mild type 1 VWD following this treatment, shifting from a qualitative to a quantitative deficiency of VWF. Considering the generally lower bleeding phenotype in type 1 VWD patients, this transition would be expected to ease the disease burden, thereby enhancing the quality of life for these patients rather than curing them entirely.

CRISPR-Cas has also been used to facilitate *in vivo* editing of murine vascular endothelium. Specifically, Cas9 was expressed under the control of the endothelial-specific CDH5 promoter from a plasmid-based expression system. Prior to retro-orbital injection, the plasmid was encapsulated in a PP/PEI (PEG_5,000_-*b*-PLGA/ PEI_25,000Da_) nanoparticle, leading to 40%–45% editing of the targeted *Pik3cg* in lung ECs of the mice, while no INDELS were detected in lung non-ECs (Zhang et al., 2022). This study shows that a specific and targeted delivery of CRISPR-Cas into ECs is possible, and the selectivity of gene targeting is highly dependent on the gRNA used. A thorough *in vitro* analysis of efficient and selective gRNAs beforehand is therefore vital and influences the editing percentage drastically.

#### 3.1.2 Homology directed repair (HDR)

While those NHEJ-based approaches are efficient, the NHEJ pathway is an error-prone repair mechanism and the outcome of the editing is not completely predictable. In contrast, HDR provides a mostly error-free repair that alters regions of DNA ranging from a few to thousand base pairs (bps) (Liao et al., 2024; Martin et al., 2019). While HDR provides a more controlled way of editing, efficiency is greatly reduced compared to NHEJ. But the approach can be highly tailored, e.g., by customization of HAs including varying lengths, and in the case of single-stranded DNA (ssDNA) templates, asymmetrical HAs (Di Stazio et al., 2021). The inclusion of truncated gRNA protospacer sequences on the HAs was also shown to aid HDR rates, the rationale being that Cas9 can bind but not cleave the HDR donor; allowing it to traffic the donor into nucleus due to the Cas9 nuclear localization signal(s) (NLS) (Nguyen et al., 2019). There are additional strategies aimed at boosting HDR efficiencies, as greatly summarized by Nambiar et al. (2022). For research purposes, cell synchronization agents and NHEJ inhibitor agents have been applied (Eghbalsaied and Kues, 2023; Selvaraj et al., 2024). Whilst they can improve HDR rates, they are toxic and not viable for therapeutic applications. A recent study on AZD7648, which inhibits NHEJ, resulted in increased translocations and large megabase deletions, including loss of chromosome arms (Cullot et al., 2024). However, chromosome rearrangements also occur spontaneously during HDR due to the involvement of different pathways managing the pairing of homologous sequences or a prematurely terminated HDR repair (Al-Zain and Symington, 2021). A major pitfall of CRISPR-Cas HDR technology is however its restriction to the S and G2 phases, where normally sister chromatids act as repair templates. Due to this, HDR is restricted to dividing cells and might therefore not be the optimal choice to edit the endothelium *in vivo*. Another limitation of inserting the full-length VWF coding sequence (∼8.4 kb) is the large payload size required, which can reduce the efficiency of *in vivo* delivery. Targeting specific regions with shorter donor templates would likely be more beneficial in terms of insertion efficiency. In VWD, the exon 4 and 5 deletion has been described in a multitude of VWD patients and would require a considerably shorter template (Bowman et al., 2012; Sutherland et al., 2009). Of course, the trade off from a targeted exon approach such as this sacrifices a broader VWD patient pool that can be treated for a smaller pool with a potential higher correction achieved.

#### 3.1.3 Homology-independent targeted integration (HITI)

Homology-independent targeted integration (HITI), as deployed by Suzuki et al., represents a strategy to increase the efficiency of gene insertions by leveraging the NHEJ pathway for donor DNA integration (Suzuki et al., 2016). This approach aims to overcome the limitations of HDR, which is often inefficient, especially in non-dividing cells. Instead of relying on HA, the donor DNA contains sgRNA binding sites located at one or both sides of the desired insert in an inverted polarity. These sites, when targeted by Cas9, can lead to the linearization of the donor template and its subsequent integration into the host genome at a CRISPR-Cas9 facilitated DSB. The reliance of HITI on NHEJ presents another limitation, as the occurrence of INDELs cannot be ruled out and a small risk of undesired fragments and inverted integration remains. Although HITI demonstrates high efficiency, particularly in non-dividing cells, its general application to VWD - like HDR - requires the delivery and integration of the full VWF coding sequence (CDS). The large size of this insert likely limits the overall efficacy of this approach. Nevertheless, HITI would present an attractive personalized editing strategy for VWD patients harboring exon deletions on *VWF,* as it has been shown to achieve precise DNA knock--in in various genetic diseases including BCD patient-derived iPSCs and mice, a hemophilia B rat model, and primary CD34^+^ HSPCs (Bloomer et al., 2020; Chen X. et al., 2022; Meng et al., 2024).

### 3.2 Base editing technology

DSDBs have been linked to undesired outcomes, such as translocations, complex mixtures of products and inactivation of p53 (Haapaniemi et al., 2018; Ihry et al., 2018; Kosicki et al., 2018). CRISPR-Cas base editing is an attractive gene editing technology due to its capability to modify nucleotides without inducing DSDBs, although unintended DSDBs have been reported in some cases resulting in genotoxic effects (Fiumara et al., 2024; Huang M. et al., 2024). The system is based on a deaminase fused to a (partially) inactive Cas protein. Numerous base editors have been developed to date, presenting variable efficiency, purity, editing window, neighboring nucleotide preference, specificity, targetability and size.

The two main types of base editors are adenine base editors (ABEs) and cytosine base editors (CBEs). ABEs convert adenine to inosine, which is read as guanine during DNA replication or repair. ABEs contain a laboratory-evolved tRNA adenosine deaminase A (TadA) for the conversion (Gaudelli et al., 2017; Shi et al., 2016). A recent ABE variant, ABE9, presents high efficiency, specificity and acts within a window of 1–2 bp (Chen L. et al., 2022). Moreover, Qin et al. developed a range of highly efficient ABE-Ultramax base editors (ABE-Umax) possessing either a wider and flexible editing window or a narrow editing window of 1–2 bp with increased specificity (Qin et al., 2024).

CBEs convert cytosine to uracil, which is read as thymine during DNA replication or repair. To prevent uracil-DNA glycosylase-mediated excision of the uracil, at least one uracil glycosylase inhibitor (UGI) is usually fused to the CBEs. The inclusion of multiple UGIs has been shown to increase efficiency, but also toxicity. Hence, limiting the number of UGIs for *in vivo* gene editing is critical. The most widely used CBE systems contain the apolipoprotein B mRNA editing catalytic polypeptide-like (APOBEC) enzymes, the *Petromyzon marinus* cytidine deaminase 1 (PmCDA1) and engineered variants thereof. However, the newest CBEs evolved from the TadA from ABEs, decreasing off-targeting probability (Lam et al., 2023).

Except from ABEs (A>G) and CBEs (C>T), additional base editors, such as CGBEs (C>G), AYBEs (A>C or A>T), gTBEs (T>C or T>G), gCBE (C>G), CABEs (C>A) and DBEs (C>T and A>G), have been developed, further expanding the applicability of the base editing technology (Chen L. et al., 2021; Kurt et al., 2020; Lam et al., 2023; Sakata et al., 2020; Tong et al., 2023; 2024). However, when compared to ABEs and CBEs, they currently exhibit lower efficiency and purity of conversion as well as larger window of activity, limiting their use in therapeutic applications. Recent reviews by Xu et al. and Wang et al. provide a very nice summary on the use of different classes of base-editors for precision medicine (Wang D. et al., 2024; Xu F. et al., 2024; Xu W. et al., 2024).

In ECFCs, adenine base editing was successfully applied by Bär et al. as a means of VWD disease modelling (Bär et al., 2025). The pathogenic VWF p.M771V point mutation was installed in healthy CB-ECFCs through lentiviral delivery of the ABE8e-SpG editor. Post enrichment of puromycin selection showed on-target efficiencies of around 70% with minimal undesired bystander editing (<2%) at neighboring base pairs. The introduced patient mutation in healthy ECFCs mimicked the patient-derived ECFC phenotype, making base editing the ideal tool to study patient mutations in the original cell type and under the control of the gene’s natural promoter. Building on this concept, precise correction of pathogenic variants using base editors would be a desirable strategy for curing VWD. However, in addition to the not fully explored side effects *in vivo* and the specific genomic requirements related to the positioning of the small editing window, the size of the base editors presents a significant limitation, which we will further discuss in the section regarding delivery.

**FIGURE 5:**
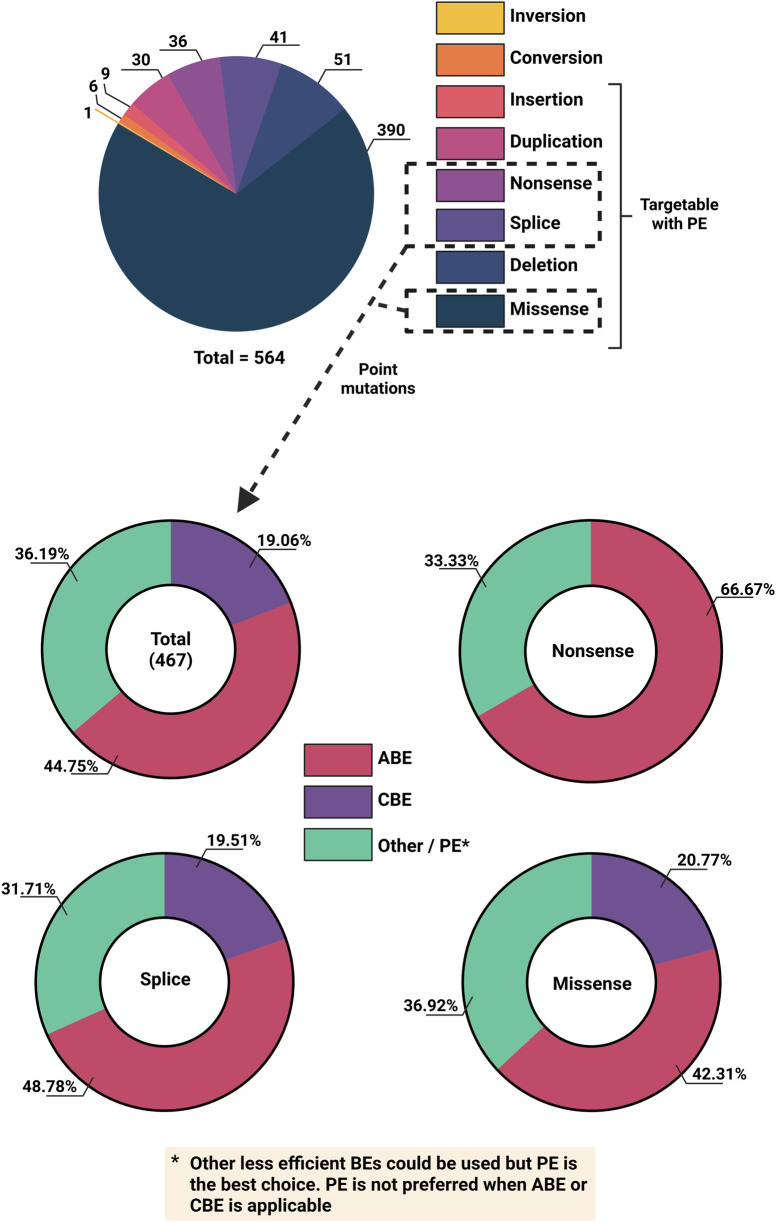
Overview of VWD mutation types and feasibility for base editing from the Leiden Open Variant Database (LOVD). Top) Number of different VWD mutation types. Bottom) Different CRISPR-Cas base editing technologies to target total Splice, Missense and non-Sense variants (and individually).

Base editors appear to be the most suitable correction tool for the majority of disease-causing variants in *VWF,* as the majority of variants are point mutations. An analysis of the LOVD database for splice, missense, and nonsense variants reveals that in principle almost half of these point mutations could theoretically be corrected with ABEs, whereas the theoretical targeting potential for CBEs lays at around 20% (Figure 5). However, the extensive heterogeneity of pathogenic VWD variants makes base editing a largely patient-specific approach, limiting its current viability due to the high costs associated with developing mutation-specific reagents. Whilst *in-vivo* clinical trials for base editing are underway, further assessment of their suitability for VWD gene therapy is required.

### 3.3 Prime editing technology

Similar to base editors, prime editors (PEs) introduce edits on target double-stranded DNA (dsDNA) without DSDBs and donor DNA. Moreover, PEs enable any type of single nucleotide modification including point mutations, small insertions, deletions and replacements, making it a potent editing tool for all the VWF variants that cannot be targeted by BEs (Figure 5). PEs consist of a partially inactive Cas9 or Cas12 protein fused to a reverse transcriptase (usually a mutant variant from Mouse-Moloney Leukemia Virus; MMLV). Instead of the standard spacer-scaffold architecture of the guide RNA, a prime editing guide RNA (pegRNA) consists of the spacer, the scaffold and additional 3′ elements, including the primer binding site (PBS) and the reverse transcription template (RTT) (Anzalone et al., 2019). When PE is guided to the target DNA loci by the spacer, it nicks the DNA, creating a 3′ DNA flap, which then hybridizes to the complementary PBS on the pegRNA. This serves as the initiation point for reverse transcription to extend the sequence using RTT, which encodes desired alterations. This ‘edited’ DNA flap can hybridize to the genome, resulting in a heteroduplex mismatch. Whether the edit is incorporated or removed depends on which strand is used for DNA repair (Anzalone et al., 2019).

Although PEs present limited off-targeting editing due to the triple checkpoint validation, they have several limiting factors, such as degradation of the 3′ end of the pegRNA and DNA mismatch repair (MMR) pathway inhibition. Several strategies have been developed to bypass these issues which have been nicely summarized by Murray et al. (2025):

- Introducing silent mutations around a desired target assists in evading the MMR pathway (Chen P. J. et al., 2021).
- Inclusion of a secondary standard 20bp nicking gRNA to nick the unedited strand, coercing the cell to utilize the edited strand as repair template. This secondary nicking gRNA can be either at the site of editing or at a short distance away (Anzalone et al., 2019).
- Fusion of a MHL1 variant with a dominant negative effect impairing MMR. MLH1 is a DNA mismatch repair protein (Chen P. J. et al., 2021).
- Addition of a 3′ motif with a strong secondary structure on the pegRNA (known as epegRNA) to prevent degradation of the 3′ PBS on the pegRNA (Nelson et al., 2022).
- Fusion of the RNA–binding exonuclease protection factor LA to the PE. LA interacts with polyuridine tracts at the 3′ end of transcripts, protecting them from exonucleases (Yan et al., 2024).

PEs have been used to target the *VEGFR-2* gene in human retinal microvascular ECs and in murine vascular ECs. Both cases achieved efficiencies just over 50% utilizing a dual lentiviral approach with a secondary nicking gRNA and MMR inhibitor (Huang X. et al., 2024; Ma et al., 2024). For applications in VWD, just like base editing, prime editing would likely fall under a personalized therapy for patient specific mutations; which currently reduces its applicability.

### 3.4 Large sequence editing: transposon and integrase-based systems

Despite the high potential of short sequence editing approaches (NHEJ, HDR, BE, PE), the large exon deletions and the high heterogenicity of pathogenic mutations in *VWF* impose the need for large sequence editors with the ability to efficiently and precisely insert or replace multiple kb in a targeted genomic site, preferably in a DSDB-free and payload size-independent manner.

Transposon systems like the Sleeping Beauty (SB) provide an avenue to deliver large transgenes such as *VWF* into the cells genome. The SB system has been utilized to transfer full length murine VWF cDNA (8.4 kb) to the liver of VWF^−/-^ mice. Whilst supra-physiological expression was stably maintained for 1.5 years, hepatocytes do not endogenously express VWF. So, the hemostatic efficacy was diminished due to reduced expression of HMW VWF multimers and did not correct the bleeding phenotype in some mice (Portier et al., 2018). Here, both episomal and random integrations into the genome were observed; highlighting the uncontrolled nature of transposons. Alternatively, Cas nucleases have been fused to transposase enzymes, generating homology-independent CRISPR transposon editors with enhanced site-specific integration. We briefly outline some of these technologies in Table 2 with references to further literature about it. Whilst these technologies, including the ones mentioned in Table 2, facilitate delivery of large transgenes such as VWF, their applicability in ECs and *in vivo* is still unknown. Despite the ability of transposon-based technologies to deliver large transgenes, their *in vivo* efficacy is currently still limited. Although there is constant progress in the development of these techniques, the on-target insertion efficiencies are overall still low. Therefore, for VWD type 1 and 2 there is an argument that this small percentage will have little impact of the bleeding phenotype and may only be viable for severe VWD type 3. Given the immature state of these techniques, we will not elaborate on them further, but Villiger et al. have discussed alternative CRISPR technologies in detail in their review, which we recommend for further reading (Villiger et al., 2024). Overall, the nascent field of large sequence editing presents several limitations, including poor characterization, low efficiency, complex design, large editor size, co-delivery of donor molecules, and undesired sequence scars. Optimization of these technologies may unlock kilobase-scale gene therapies for VWD.

**TABLE 2:** Overview of CRISPR-Cas based transposon and integrase technologies to facilitate large insertion templates.

Technology	CRISPR technology	Fusion system	Source/reference
Find and cut-and-transfer (FICAT)	Cas (DSDB generated)	PiggyBac (PB) transposase	Pallarès-Masmitjà et al. (2021)
transCRISTI	Cas (DSDB generated)	PiggyBac (PB) transposase	Bazaz et al. (2022)
Programmable addition via site-specific targeting elements (PASTE)	Prime editor	Serine integrase Bxb1	Fell et al., 2024; Yarnall et al., 2022
CRISPR-associated transposases (CASTs)	dCas	Tn7 and Tn5053 like	Strecker et al., 2019; Witte et al., 2025
prime-editing-assisted site-specific integrase gene editing (PASSIGE)	Prime editor	Serine integrase Bxb1	Pandey et al. (2024)

## 4 *In vivo* delivery systems

**FIGURE 6:**
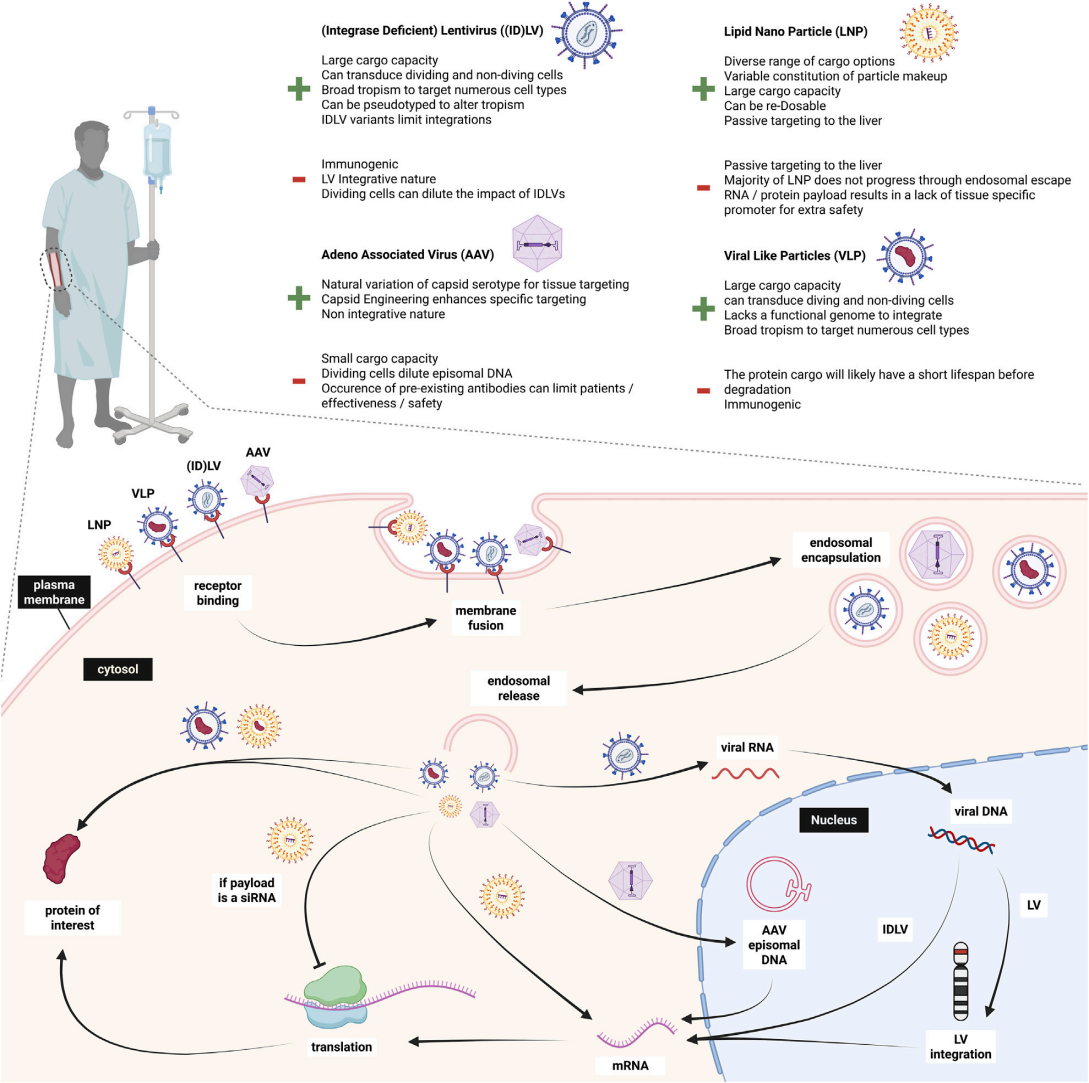
Overview of *in vivo* delivery approaches to endothelial cells and different payload mechanisms. Created in BioRender.

Delivery of gene editing systems is one of the major challenges holding back research and application of gene therapy *in vivo*. For VWD, multiple studies have been carried out in VWF^−/-^ mice through hydrodynamic injection. As mentioned above, the SB system (SB100x) successfully delivered *VWF* to the liver for prolonged ectopic expression. Another study by De Meyer et al. also demonstrated that plasmid hydrodynamic injection resulting in ectopic liver VWF expression can be sufficient to restore thrombus formation; highlighting the feasibility of VWF rescue for severe VWD *in vivo* (Meyer et al., 2008). In this section, we elaborate on different delivery systems including adeno-associated viral vectors (AAV), adenoviral vectors (Ad), retroviral vectors (RV), (integrase-deficient) lentiviral vectors ((ID)LV), lipid nanoparticles (LNPs), and virus-like particles (VLPs); most of which are summarized in Figure 6. Besides these popular delivery systems, other techniques have been explored in the last years for *in vivo* gene therapy delivery, such as cell-penetrating proteins (CPPs), extracellular vesicles (EVs), and ultrasound-mediated drug delivery (Cecchin et al., 2023; Perolina et al., 2024; Rackear et al., 2024; Yu et al., 2021). While these approaches are not new, they have undergone different modification approaches, tailoring them more towards targeted, and safe delivery. However, they still require more investigation.

With the choice of which delivery system to use also comes the decision of which endothelium to target. Since all ECs produce VWF, the preferred target location could be tailored to the delivery approach. Generally, the vascular endothelium lining the blood vessels gets easily exposed by any intravenously administered product and is the main producer of VWF. A significant contributor to VWF levels in blood is the lung endothelium (Kaufmann et al., 2000; Yamamoto et al., 1998). Those ECs could be specifically targeted in view of their large contribution to circulating VWF. A third option of targetable endothelium would be liver sinusoidal endothelial cells (LSEC) (Milani et al., 2024). As any drug, a potential gene therapeutic agent would pass the liver, and the regenerative property of the liver could add another safety measure in case of undesired outcomes (Herman and Santos, 2023). The desired target cell population therefore depends on the chosen delivery strategy and could vary in their safety and efficacy.

### 4.1 Adeno-associated viral vectors

AAVs consist of a ssDNA genome and an icosahedral protein capsid. They are attractive due to their low immunogenicity and non-integrative nature. Furthermore, the natural occurrence of various serotypes can help elude existing neutralizing antibodies; which are considered a contra-indication for most AAV gene therapies. For bleeding disorders such as hemophilia A and B, AAVs are currently being used with transgenes of factor VIII and factor IX (Blair, 2022; Chowdary et al., 2022; George et al., 2021; Heo, 2023; Ozelo et al., 2022). These studies directed AAVs to the liver to achieve prolonged factor expression. Subsequently, most patients could discontinue prophylactic use of clotting factor concentrate infusions and the number of bleeds were strongly reduced. A review by Zwi-Dantsis et al. greatly summarizes AAVs and their applications in gene therapy (Zwi-Dantsis et al., 2025). Unfortunately, the tropism of AAVs for ECs is rather low, challenging EC transduction (Sreeramoju and Libutti, 2010). This can be enhanced through capsid modification, such as peptide insertion, which can alter specificity and infectivity to specific cells. This was shown by Varadi et al. where capsid engineered AAV2 and 9 containing peptide ligands selected from random peptide libraries were successfully used to enhance transduction of different EC types, such as HUVECS (Varadi et al., 2011). Nevertheless, there are limitations including the fading therapeutic effects over the years, but one of the main limitations of AAVs with respect to VWD is their limited cargo capacity of around 4.5 Kb. Consequently, a single AAV approach is unfeasible for large transgenes such as SpCas9, LbCas12a and VWF, with a CDS length of ∼8.4 Kb.

Dual AAV systems can be applied to circumvent the size issue but add further complications: firstly, they usually require a protein fragment such as a split-intein to combine both terminals together (Wu Y. et al., 2023). This can be less efficient than standard expression. Secondly, a higher dose of AAV titer is required which can cause immunogenic complications during treatment. Finally, target cells need to be transduced by both AAV constructs to be effective. This approach was tested in HUVECs and mice, delivering a human VWF transgene split across dual AAVs utilizing a recombinant region from the AK region of the F1 phage genome (Barbon et al., 2021). The plasma levels were between 1% and 1.5% of WT VWF and determined to be subtherapeutic.

Generally, single AAV outcompetes dual AAV systems, especially when AAV dose or tissue can prevent saturating levels of transduction (Davis et al., 2022). As the field evolves, it is likely that more efficient dual AAV delivery systems will be developed; but as it stands now, it is unlikely that AAVs will be utilized for VWF gene therapy.

### 4.2 Adenoviral vectors

Ads are commonly studied as vectors for transgene delivery, oncolytic agent, and vaccines (Paul et al., 2003; Peter and Kühnel, 2020; Smaill et al., 2013). In humans, more than 57 serotypes of Ads have been found to form seven Ad groups A-G (Wold and Toth, 2013). While this offers a big variety of Ads, most people are immune to several serotypes due to previous Ad infections. Ad’s genetic material is dsDNA with a length of 25–46 kb. The cargo capacity of Ad5, the most used Ad serotype, exceeds AAVs by far with 8–36 kb compared to AAV’s ∼5 kb (Shiver et al., 2002). Engineered Ad5 has been shown to transduce the vascular endothelium and ECs across multiple organs in mice (Lu et al., 2014). While they have many advantages such as their capacity to infect non-dividing and dividing cells of various tissues, their non-integrative nature and large cargo, the strong host immunogenicity and cellular toxicity of Ad vectors remain a major problem for their use in gene therapy (Wang and Shao, 2023).

### 4.3 Retroviral vectors

The first human cancer gene therapy approaches were facilitated with retroviruses, which consist of three subcategories: gamma-retroviruses, alpha-retroviruses, and lentiviruses. They all possess the ability to randomly integrate their cargo into the host-cell genome, thereby imposing a higher safety concern when compared to non-integrative gene therapy platforms (Shao et al., 2022). Liu et al. used a retroviral approach to target tumor-associated ECs and developed a successful transduction system of HUVECs and KSY1 ECs (Liu et al., 2000). However, the adverse events of secondary cancer developments, including leukemia, render retroviruses too dangerous to use in the clinic (Li et al., 2002).

As one of the most used retroviruses in research, we only further elaborate on the lentiviral system.

### 4.4 Lentiviral vectors

LV systems have large size capacities of around 9 Kb. This makes them attractive for delivery of large transgenes and led to the FDA approval of several LV-based gene therapies in the last years (Ali et al., 2019; Jensen et al., 2021; Keam, 2021; Schuessler-Lenz et al., 2019). LVs infect dividing and non-dividing cells–in contrast to gamma-retroviral vectors solely infecting dividing cells–which would make LVs attractive to target ECs, but low transduction efficiency and significant vector-associated cytotoxicity has been reported for ECs (Sreeramoju and Libutti, 2010). *In vivo* pseudotyping of LVs is pivotal to assure efficient and predominant delivery of agents to desired cells/tissues. The VSV-G pseudotype has successfully delivered Cas9 to CB-ECFCs and canine venous blood-derived ECs *ex vivo* (Meyer et al., 2006; Schillemans et al., 2019). In a proof of principle study, phenotypic correction of canine VWD ECFCs was obtained by LV-mediated gene delivery (Meyer et al., 2006). The GP64 pseudotyped LV vector has been shown to transduce liver sinusoidal endothelial cells (LSECs) more efficiently than vascular ECs. This was taken further with FVIII delivery to LSECs in FVIII KO mice (Milani et al., 2024). Other *in vivo* studies showed benefits of HIV-derived LV vectors over AAV vectors due to the decreased prevalence of HIV-directed antibodies in humans, reducing the host immune response (Follenzi et al., 2007). LV delivery was also explored in a canine model to treat hemophilia B with surprisingly positive results and no long-term toxicity in the animal model (Cantore et al., 2015). LV delivery was used to ensure stable gene integration in the liver cells, preventing dilution of the factor IX transgene. Common insertion sites (CIS) have been identified in patients undergoing LV gene therapy for adrenoleukodystrophy (ALD). Biffi et al. demonstrated that these CIS arise from a benign integration bias rather than oncogenic selection (Biffi et al., 2011). However, a recent study using LVs in ALD reported the development of hematological cancers due to clonal vector insertions within oncogenes (Duncan et al., 2024).

To increase the safety profile of LVs, IDLVs have been developed. The vector DNA of IDLV exists as non-replicating episomal DNA which prevents integration (Cortijo-Gutiérrez et al., 2021). Nevertheless, the episomal DNA can be subjected to epigenetic silencing, limiting the efficacy of treatment (Suwanmanee et al., 2014). Furthermore, unintegrated DNA is diluted out through cell division, so safe delivery of transiently acting proteins or protein fusions may be feasible using IDLVs (Wanisch and Yáñez-Muñoz, 2009).

### 4.5 Lipid nanoparticles (LNPs)

To date, LNPs are the most popular non-viral delivery systems, as they are being developed and optimized since the 1990s. They are FDA-approved (COVID-19 Pfizer/BioNTech and Moderna vaccines) and they exhibit low immunogenicity, transient expression and feasibility for large-scale production (Jung et al., 2022). Generally, LNPs are mainly used to deliver RNAs (siRNA or mRNA) but have also been shown to deliver CRISPR-Cas RNPs (Chen et al., 2024). The natural target site of LNPs are liver hepatocytes due to their neutral charge and the spontaneous binding of apolipoprotein E (ApoE) that functions as an endogenous ligand for hepatocytes. This property has been exploited for the development of LNP-based gene editing approaches for treatment of transthyretin amyloidosis as well as hereditary angioedema (Cohn et al., 2024; Gillmore et al., 2021). Interestingly, depending on their specific composition, some LNPs accumulate in LSECs, which leads to immune responses according to Sato et al. (2017). While this can be circumvented by reengineering the LNP composition, it can also be used to specifically target LSECs by modifying physical size and incorporating active targeting ligands, such as mannose or antibody moieties, to the LNP (Campbell et al., 2018; Kim et al., 2021; Pattipeiluhu et al., 2022). A recent review by Wang et al. provides a comprehensive overview of LNPs focusing on mRNA delivery, therapeutic applications and targeting mechanisms (Wang J. et al., 2024).

### 4.6 Viral-like particles (VLPs)

VLPs may provide an alternative to traditional LV approaches by combining the benefits of viral and non-viral delivery. VLPs are generated utilizing a fusion of the transgene of interest to the LV matrix gag protein. The most popular VLPs are pseudotyped with VSV-G glycoprotein, HIV-1 envelope glycoprotein and so-called ‘nanoblades’, which are murine leukemia VLPs loaded with Cas9-sgRNA ribonucleoproteins (RNPs) (Mangeot et al., 2019). Essentially a LV capsid is produced encapsulating the protein of interest, but while the envelope and capsids are viral components, the genome is non-viral. The cargo of mRNA, proteins or RNPs increase the safety by staying transiently expressed instead of integrating into the host genome. This improves CRISPR-Cas therapies regarding off-target activity and host immune response against the Cas protein, while still facilitating efficient genome editing (Lyu et al., 2019). The group of David Liu have engineered VLPs specifically to deliver BEs and PEs, which renders them promising delivery tools *in vivo* (An et al., 2024; Banskota et al., 2022). Furthermore, VLPs are relatively cost-effective and easily produced but the batch variability remains problematic (Jaron et al., 2022). Moreover, the immune response and rapid clearance of VLPs *in vivo* remains a potential concern. For further reading on this topic, see the reviews from Gupta et al. (2023), Nooraei et al. (2021), and Tariq et al. (2022).

As of now, with fine tuning of capsids, lipid composition, and pseudtyping, an array of delivery vehicles can be directed to the targeting of EC’s *in-vivo*. Smaller platforms such as AAVs that cannot accommodate the bulky nature of gene correction technologies and VWF currently sit in a suboptimal position; despite their attractive non-integrative nature. This likely leaves non integrative systems than can package larger loads as the foremost options.

## 5 Is gene therapy the future of VWD treatment?

While the demand for new VWD treatments is clear, the path to achieving them remains uncertain. Exploiting the latest advances in genome editing and gene delivery mentioned in this review, targeting the root cause of VWD through gene therapy now appears increasingly feasible.

Gene therapy can be achieved through either an *ex vivo* (manipulation of cells outside of the body) or an *in vivo* (direct manipulation inside the body) approach. As the predominant producers of VWF, ECs would be the optimal candidate for targeted therapy. Furthermore, ECs line the vasculature and therefore would be readily exposed to any agents delivered intravenously. The most common and accessible patient-derived ECs are venous blood-derived ECFCs. These cells are hampered by limited growth potential *in vitro*, and a vast heterogeneity between donors in terms of growth and isolation success, which could make *ex vivo* correction and transplantation problematic (Olgasi et al., 2021). Alternatively, human induced pluripotent stem cells (hiPSCs) derived from the patient as an autologous source could give rise to ECFCs through the mesoderm lineage, and multiple studies have successfully produced ECFCs *in vitro* (Abutaleb and George, 2021; Hamad et al., 2022). However, these models often exhibit a more embryonic state compared to primary ECFCs, and as such often lack WPBs, or produce pseudo-WPBs rather than the clear elongated shape that is expected (de Boer et al., 2024). These phenotypical discrepancies are too severe to use hiPSCs-derived ECs for VWD therapy and their use is even critically discussed for *in vitro* research. Consequently, *in vivo* gene therapy is the only feasible option for VWD gene therapy.

The allele-selective KO approach using siRNA that was discussed above seems to be one of the most promising approaches to alleviate VWD *in vivo*. So far, mouse models show impressive rescue of the bleeding phenotype but the duration of the siRNA approach and the feasibility of repeated treatment still needs further investigation (Jongejan et al., 2023). *In vivo* delivery of therapeutic siRNA was achieved employing encapsulation into 7C1 oligomeric lipid nanoparticles (composed of PEG, cholesterol and dioleoylphosphatidylethanolamine) specifically containing C14 alkyl PEG moieties and were shown to primarily target ECs (Sago et al., 2018; Yu et al., 2019). The targeting ability of 7C1-containing LNPs paves the way for siRNA- or CRISPR-based allele-selective disruption of mutant VWF alleles in VWD patients carrying dominant negative heterozygous mutations on their *VWF*. Furthermore, these studies provide a basis for the cutting-edge base and prime editing technologies, especially these composed of compact Cas proteins, that can be tailored to a variety of mutation types.

Proof-of-principle for the feasibility of gene editing employing a lentiviral-based CRISPR-Cas9 ABE in cord blood derived ECs has been shown with the goal of introducing a suspected pathogenic mutation in exon 18 of VWF (Bär et al., 2025). This observation provides an avenue towards *in vivo* correction of VWD employing CRISPR-Cas9 base and prime editing strategies. First *in vitro* experiments using a CBE on patient-derived ECFCs to correct the patient mutation p.M771V showed that correction of at least one allele leads to a rescue of the ECFC phenotype. However, in the current state efficiency of this method remains too low (<6%) to translate the genetic correction to a rescue of the bleeding phenotype. In addition, the low proliferative nature of ECs *in vivo* excludes most of the other genetic tools and enhances the risks associated with stable CRISPR-Cas expression in the cells. Hence, non-viral delivery of base and prime editors using the aforementioned 7C1-containing LNPs would prevent continuous editing and thus limit off-target effects that would most likely result from viral delivery systems.

Another point to consider for VWD gene therapy is the huge variety of pathogenic variants along *VWF*. Gene correction therapy needs to be personalized, which currently is associated with high costs. However, targeting the endothelium seems to be a durable approach and could potentially alleviate patient burden for several years.

Taking everything into consideration, considerable progress has been made with respect to the development of novel approaches for VWD gene therapy. *In vivo* delivery of siRNA-loaded LNPs targeting ECs has emerged as a potential treatment strategy for dominant negative VWD type 2. *In vitro* evidence for base editing approaches in ECFCs has recently been obtained. We anticipate that continuously evolving editing systems will allow for the generation of highly specific on-target editing tools with low off-target effects. The simultaneous evolution of delivery systems that can efficiently target therapeutic cargo to ECs has the potential to launch a new era of gene therapy for patients suffering from VWD.

## Glossary

(ID)LV: (integrase-deficient) lentiviral vector
AAV: Adeno-associated viral vector
ABE/CBE: Adenine base editor/cytosine base editor
Ad: Adenoviral vector
Cas: CRISPR-associated
CAST: CRISPR-associated transposase
CB: Cord blood
CDS: Coding DNA sequence
CIS: Common integration site
CPP: Cell-penetrating protein
CRISPR: Clustered regularly interspaced short palindromic repeats
DDAVP: Desmopressin
DSDB: Double strand DNA break
EC: Endothelial cell
ECFC: Endothelial colony forming cell
ER: Endoplasmic reticulum
EV: Extracellular vesicle
FiCAT: Find and cut and transfer
HA: Homology arm
HDR: Homology-directed repair
hiPSC: Human induced pluripotent stem cell
HITI: Homology-independent targeted integration
HMW: High molecular weight
INDELs: Insertions and/or deletions
LMW: Low molecular weight
LNP: Lipid nanoparticle
NHEJ: Non-homologous end joining
PAM: Protospacer adjacent motif
PASTE: Programmable addition via site-specific targeting elements
PE: Prime editor
RV: Retroviral vector
TALEN: Transcription activator-like effector nucleases
TransCRISTI: Transposase-CRISPR mediated targeted integration
UL: Ultra large
VLPs: Viral-like particle
VUS: Variants of uncertain significance
VWD: Von Willebrand disease
VWF: Von Willebrand factor
VWFpp: Von Willebrand factor propeptide
WPB: Weibel Palade Bodie
ZFN: Zinc-finger nuclease
