# Correction: Rewriting the script: gene therapy and genome editing for von Willebrand Disease

**DOI:** 10.3389/fgeed.2025.1719330

**Published:** 2025-11-07

**Authors:** Alastair Barraclough, Isabel Bär, Tirsa van Duijl, Karin Fijnvandraat, Jeroen C. J. Eikenboom, Frank W. G. Leebeek, Ruben Bierings, Jan Voorberg, Despoina Trasanidou

**Affiliations:** 1 Department of Pediatric Hematology, Emma Children’s Hospital, Amsterdam UMC, University of Amsterdam, Amsterdam, Netherlands; 2 Department of Hematology, Erasmus University Medical Centre, Rotterdam, Netherlands; 3 Molecular Hematology, Sanquin Research and Landsteiner Laboratory, Amsterdam University Medical Centre, Amsterdam, Netherlands; 4 Division of Thrombosis and Hemostasis, Department of Internal Medicine, Leiden University Medical Centre, Leiden, Netherlands

**Keywords:** gene therapy, VWD, endothelial cells, delivery, CRISPR-Cas, *in vivo* delivery

There was a mistake in the caption of Figure 1 as published. The legend of Figure 1 included text from an older version of the figure, which should have been removed. The incorrect caption read: Overview of von Willebrand Disease sub-types, von Willbrand Factor (VWF) domains, and variant distributions. **(A)** Summary of von Willebrand Disease sub-types **(A)** Von Willebrand Factor domains and binding sites. **(B)** Distribution and frequency of von Willebrand Factor variants curated in the Leiden Open Variation Database (LOVD) (Accessed and data retrieved on 8-1-2025). Created in BioRender.

The corrected caption of Figure 1 appears below.

Overview of von Willbrand Factor (VWF) domains, and variant distributions. **(A)** Von Willebrand Factor domains and binding sites. **(B)** Distribution and frequency of von Willebrand Factor variants curated in the Leiden Open Variation Database (LOVD) (Accessed and data retrieved on 8-1-2025). Created in BioRender.

The original article has been updated.

